# Risk Factors, Diagnosis, and Treatment of Neonatal Fungal Liver Abscess: A Systematic Review of the Literature

**DOI:** 10.3390/life13010167

**Published:** 2023-01-06

**Authors:** Paschalia Kopanou Taliaka, Andreas G. Tsantes, Aikaterini Konstantinidi, Dimitra Houhoula, Konstantina A. Tsante, Aristeidis G. Vaiopoulos, Daniele Piovani, Georgios K. Nikolopoulos, Stefanos Bonovas, Nicoletta Iacovidou, Argirios E. Tsantes, Rozeta Sokou

**Affiliations:** 1 Neonatal Intensive Care Unit, “Agios Panteleimon” General Hospital of Nikea, 18454 Piraeus, Greece; 2 Microbiology Department, “Saint Savvas” Oncology Hospital, 11522 Athens, Greece; 3 Department of Food Science and Technology, University of West Attica, 12244 Egaleo, Greece; 4 Laboratory of Reliability and Quality Control in Laboratory Hematology, Department of Biomedical Science, School of Health and Caring Science, University of West Attica, Egaleo, 12244 Athens, Greece; 5 Laboratory of Haematology and Blood Bank Unit, “Attiko” Hospital, School of Medicine, National and Kapodistrian University of Athens, 12462 Athens, Greece; 6 IRCCS Humanitas Research Hospital, Via Manzoni 56, Rozzano, 20089 Milan, Italy; 7 Department of Biomedical Sciences, Humanitas University, Pieve Emanuele, 20090 Milan, Italy; 8 Medical School, University of Cyprus, Nicosia 2029, Cyprus; 9 Neonatal Department, National and Kapodistrian University of Athens, Aretaieio Hospital, 11528 Athens, Greece

**Keywords:** fungal infection, liver abscess, neonates, candidemia, preterm, antifungal therapy

## Abstract

(1) Background: Although invasive fungal infections are a major cause of neonatal morbidity and mortality, data on the incidence and outcomes of localized abscesses in solid organs due to fungal infections are scarce. The aim of this study was to consolidate evidence and enhance our understanding on neonatal liver abscesses due to invasive fungal infections. (2) Methods: An electronic search of the PubMed and Scopus databases was conducted, considering studies that evaluated fungal liver abscesses in the neonatal population. Data on the epidemiology, clinical course, treatment, and outcome of these infections were integrated in our study. (3) Results: Overall, 10 studies were included presenting data on 19 cases of neonatal fungal liver abscesses. *Candida* spp. were the most common causative pathogens (94.7%). Premature neonates constituted the majority of cases (93%), while umbilical venous catheter placement, broad spectrum antibiotics, and prolonged parenteral nutrition administration were identified as other common predisposing factors. Diagnosis was established primarily by abdominal ultrasonography. Medical therapy with antifungal agents was the mainstay of treatment, with Amphotericin B being the most common agent (47%). Abscess drainage was required in four cases (21%). Eradication of the infection was achieved in the majority of cases (80%). (4) Conclusions: Even though fungal liver abscess is a rare entity in the neonatal population, clinicians should keep it in mind in small, premature infants who fail to respond to conventional treatment for sepsis, particularly if an indwelling catheter is in situ. A high index of suspicion is necessary in order to achieve a timely diagnosis and the initiation of the appropriate treatment.

## 1. Introduction

Invasive fungal infections (IFI) are a major cause of neonatal morbidity and mortality, affecting mainly preterm and very low birth weight (VLBW) neonates (<1500 g). Candida species are responsible for the vast majority of IFI in this age population, with a reported incidence of 5–10 cases per 100,000 live-born infants [1]. Other fungal species such as Malassezia, Aspergillus, and Zygomycetes contribute to a lesser degree to the overall disease burden [2].

A host of unique predisposing factors such as immature immune function and host epithelial protection, hospitalization in an intensive care setting, and frequent invasive procedures render neonates particularly vulnerable to infectious insults. The incidence of invasive candidiasis (IC) is inversely related to the gestational age and birth weight (BW), rising from 0.06% in hospitalized neonates with a BW > 1500 g to 2–5% in VLBW infants [3]. The spectrum of clinical manifestations of Candida infection in neonates includes bloodstream and urinary tract infections, meningitis, endophthalmitis, endocarditis, osteomyelitis, and the localized abscesses of solid organs.

Neonatal liver abscess (LA) is a rare but life-threatening entity, associated with a high mortality rate. Since 1930, less than 150 cases have been reported in the literature [4,5,6,7]. The increased survival rate of extremely premature babies due to advances in neonatal care, and the wide use of bedside imaging modalities such as portable ultrasound, have led to an increased rate in the reporting of this entity over the last few decades. The most common pathogens accounting for neonatal LA are *Staphylococcus aureus*, *Streptococcus pyogenes*, *Escherichia coli*, *Klebsiella* spp., and *Pseudomonas* spp. [8]. However, data on the incidence, clinical course, and outcome of fungal liver abscess in the neonatal population are scarce.

The aim of this systematic review was to consolidate evidence and enhance our understanding on neonatal fungal liver abscesses regarding the microbiology, risk factors, clinical course, diagnosis, and treatment of these infections.

## 2. Materials and Methods

### 2.1. Search Protocol/Databases

A methodological protocol was developed based on the guidelines of the Preferred Reported Items for Systematic Reviews and Metanalysis (PRISMA, presented as a Supplementary Material) in order to identify and assess studies relevant to the topic of interest of this review [9,10]. The review of the literature was conducted between October 2022 and November 2022. The systematic review was not registered in Prospero.

An electronic search of the existing literature in PubMed and Scopus databases until 3 November 2022 was conducted using combinations of the following keywords: “fungal”, “candida”, “liver abscess”, “hepatic abscess”, “newborn*”, “neonate*”, and “preterm neonate*”, with Boolean logic operators. Duplicate records were removed by a single researcher (R.S.) using the default settings of EndNote X8 software. Initially, two independent researchers (R.S. and P.K.T.) individually screened the titles and abstracts, and clearly irrelevant articles were excluded. A thorough full-text evaluation was followed by the researchers, resulting in the inclusion of only those studies that met the established inclusion criteria. Disagreements between the two researchers regarding the inclusion of studies were resolved with the contribution of a third author (A.G.T.). Additionally, in order to minimize the possibility of missing any relevant studies, a manual search of the electronic records was performed, while the citations of each retrieved study and of previous systematic reviews pertaining to our topic of interest were also screened.

### 2.2. Study Selection and Data Extraction

Randomized clinical trials, observational studies, case series, and case reports involving neonatal patients with a documentation of fungal liver abscess were considered eligible for this review. Narrative reviews, systematic reviews, and/or meta-analysis were excluded, as well as studies published in languages other than English. No geographic restrictions were applied. After compiling the relevant studies, the data of interest for each study were extracted into an excel file. The extracted data for each study included: the first author, year of publication or presentation, number of participants, participant characteristics and underlying condition, information on the treatment, complications, and the outcome.

## 3. Results

**Figure 1:**
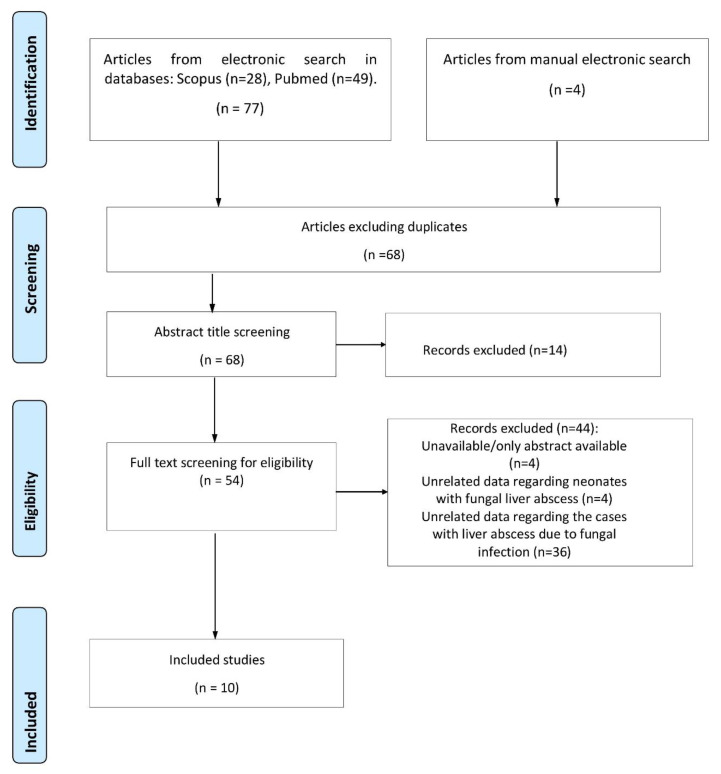
Flow chart of study selection process.

Our systematic review yielded a total of 77 studies. After a thorough evaluation, 10 studies describing 19 patients met the inclusion criteria and were incorporated in our study. Detailed information on the study selection process is presented in Figure 1.

Detailed data for each patient regarding the demographics, clinical presentation, treatment, and outcome of the infection are presented in Table 1.

The majority of the included neonates were preterm (14/15 neonates with a reported gestational age, 93%), with a median gestational age of 29 weeks (range 24 weeks to term). The median patient age at the presentation was 22.5 (range 7–59) days (Table 2).

A male predominance 2:1 was shown in studies reporting data on the patient’s sex (eight male vs. four female neonates). Besides prematurity, several other predisposing factors were identified, including total parenteral nutrition (TPN) administration (12/13 neonates, 92.3%), umbilical venous catheter placement (13/13 cases, 100%), mechanical ventilation (7/8 neonates, 87.5%), and previous broad-spectrum antibiotic administration (11/12 cases, 91.7%). Four infants (21%) had a history of a previous surgical abdominal intervention; two due to necrotizing enterocolitis (NEC), one due to duodenal atresia, and one due to ileal atresia. None of the neonates had previously received antifungal prophylaxis.

*Candida* spp. were the most commonly isolated pathogens (isolated in 94.7% of cases), with *Candida albicans* accounting for 11/19 cases (57.9%), *Candida parapsilosis* for 1 (5.2%) case, *Candida glabrata* for 1 (5.2%) case, and unidentified Candida species for 5/19 cases (26.3%). Only one case of a non-Candida pathogen (Malassezia) was reported (Table 3).

Fungi were isolated from blood cultures in 17/19 (89.5%) cases, from fluid aspirated from the abscess in 8/19 (42.1%) cases, and from cerebrospinal fluid in 2/19 (10.5%) cases (Table 4). In one case, even though no pathogen was cultured in blood and abscess fluid, a microscopic examination of the abscess fluid (stained with calcofluor white) revealed hyphae and yeast-like cells indicative of Malassezia [18].

A clinical presentation consistent with sepsis (often with a prolonged course) was reported in most cases, whereas other symptoms such as liver enlargement were reported in some neonates. The abscess was identified on abdominal ultrasonography (U/S) in all cases except for one, in which the abscess was discovered during abdominal surgery; the relevant data were not available for four of the cases. Four neonates with positive findings on U/S underwent abdominal computed tomography (CT) for a further evaluation. Additional lesions in organs other than the liver were present in four cases; a brain abscess in two cases, a subphrenic abscess in one case, and a gallbladder lesion (attributed to the rupture of the abscess into the biliary tree) in another case. Concomitant infection with another microorganism was also reported in four cases, involving *Acinetobacter baumanii*, *Klebsiella* spp., methicillin-resistant *Staphylococcus aureus,* and *Xanthomonas maltophilia* (Table 3). The abscesses were located predominantly in the right lobe (eight in the right lobe vs. three in the left lobe; the location of the abscess in the remaining cases was not specified). The majority involved one solitary lesion (8/12 neonates, 66.7%), whereas multiple abscesses were reported in 4 (33.3%) neonates; no data were available for the remaining cases (Table 4).

The antifungal therapy differed significantly among the cases, with amphotericin B (AMB) being the most commonly used agent. Monotherapy with different formulations of AMB (liposomal, deoxycholate, or unspecified) was reported in 8 out of the 15 cases with available data on the administered antifungal agents (53%). Other treatment regimens included a combination of AMB and flucytosine (1/15 neonates, 6.6%), fluconazole and subsequently AMB (1/15 neonates, 6.6%), monotherapy with micafungin (1/15 neonates, 6.6%), fluconazole followed by caspofungin (1/15 neonates, 6.6%), and a combination of fluconazole, AMB, and caspofungin (1/15 neonates, 6.6%). In one noteworthy case, AMB was injected inside the abscess in addition to the intravenous administration of micafungin and AMB, leading to resolution of the infection. The median duration of an antifungal administration (excluding infants who expired) was 34 days (range 28–42). Additional invasive treatment was required in 4/19 cases (21%), including the placement of a percutaneous pigtail catheter in 3 cases and open surgical drainage in 1 case. The infection resolved in the majority of cases (12/15 neonates, 80%) and only 3 (20%) neonates died; data on the outcome were not available in 4 cases (Table 5).

## 4. Discussion

This systematic review investigated the existing literature regarding fungal liver abscess in the neonatal population. *Candida* spp. were identified as the most common causative pathogens (94.7%), while prematurity, broad-spectrum antibiotic administration, umbilical vein catheterization, and prolonged parenteral nutrition were identified as common predisposing factors. The presenting symptoms were often vague and non-specific, but the majority of neonates exhibited clinical manifestations consistent with sepsis. Conservative therapy with antifungal agents was the mainstay of treatment, with AMB being the most common agent (47%), while abscess drainage was required in four cases (21%). The infection was resolved in the majority of the studied cases (80%).

Neonatal IFI are life-threatening opportunistic infections, reported as the third most common cause for late-onset sepsis in VLBW infants. The rate of these infections varies greatly among different settings, with a reported median rate of 7.5% in ELBW neonates [20]. Candida species are responsible for the vast majority of IFI, with *C. albicans* accounting for the majority of cases (60–75%), followed by *C. parapsilosis* (20–30%), *C. krusei,* and other non-*albicans* species [1]. *C. auris* is an emerging pathogen that has caused significant concern worldwide since it was first described in 2009 due to its resistance to antifungal therapy and reported high mortality rate. Data on its incidence in the neonatal population are sparse with only a few reported cases, but the real impact could be underestimated due to misidentification by conventional microbiological assays [21]. The risk of IFI is higher in low-weight infants, with 86% of all cases occurring in neonates under 1000 g, while the mortality rate ranges from 25 to 55% [22,23]. Moreover, neonates who overcome these infections commonly present with late sequelae such as hearing or/and visual impairment, cerebral palsy, and mental retardation [24,25]. In 2004, Stoll et al. reported that neurodevelopmental impairment was observed during follow-up in 57% of ELBW neonates that survived IFI [25]. Timely diagnosis is extremely challenging as symptoms can be subtle and clinical presentation may be indistinguishable from other infections.

LA in neonates are rare entities and often present as a complication of sepsis, NEC, or surgical abdomen. Diagnosis is elusive as the symptoms are vague and non-specific. Laboratory tests usually include elevated infection markers and transaminases [7]. Four possible mechanisms of pathogen invasion into the liver and subsequent abscess formation are described in the literature: (a) hematogenous spread, (b) ascending infection through the umbilical and portal veins or (c) the biliary tree, and (d) contiguous spread from the adjacent structures [4,7,8].

Very few cases of fungal LA have been reported in the literature. Our results indicate that preterm neonates are most affected by these infections (93% of all cases) since the immaturity of the innate immune system and underdeveloped physical barriers render them at a higher risk for invasive infections. Skin or mucosal colonization with fungal pathogens during hospitalization is a recognized risk factor for the later development of IFI. Neonates with candidemia are reportedly six times more likely to have previously experienced a colonization by the same species [26]. About 88% of fungal colonizations are reported to occur during the first days of life, in sites such as the anus, oral cavity, and umbilicus [27]. A colonized skin and GI tract can become the primary source of the translocation of pathogens through damaged or compromised epithelium and mucosa, respectively, leading to the systemic dissemination of these pathogens [28,29]. The administration of broad-spectrum antibiotics causes alterations in the normal intestinal microbiota and facilitates the invasion by pathogenic species in the setting of a prolonged NICU hospitalization [30]. In line with this, most neonates in our study had previously received broad-spectrum antibiotics.

Abdominal instrumentation and indwelling devices, particularly umbilical venous catheters (UVC), have been identified as important risk factors for the development of LA. Due to the unique anatomy of umbilical vessels and the presence of patent ductus venosus in neonates, the UVC tip can migrate and subsequently lie inside the liver. Moreover, the use of a mal-positioned UVC to infuse hypertonic solutions, such as parenteral nutrition and lipids, can lead to topical necrosis of the hepatic tissue, creating ideal conditions for the formation of an abscess [31]. The association between UVC placement and LA formation is also supported by the results of our study, with 13 of our cases concerning infants who had a history of UVC placement.

An intriguing finding was that four of the patients included in our study exhibited concurrent infection with fungal and bacterial pathogens. This condition has been extensively studied during the last years in adult populations, with various reports raising the percentage of bacteremia in hospitalized patients with candidemia to 27–67% [32,33]. The hypothesis that fungal infection carries an increased risk of bacterial coinfection could have serious implications on the treatment choices and management of neonates with fungal liver abscess.

Abscesses located in other organs, most notably the brain, are often discovered in neonates initially diagnosed with LA, a fact mirrored by the data in our study. The underlying pathophysiological mechanism is thought to be through metastatic septic emboli in the circulation. In a review by Benjamin et al., end-organ damage in neonates with candidemia was reported with a median prevalence of endophthalmitis 3%, meningitis 16%, ventriculitis/brain abscess 4%, endocarditis 5%, renal abscess 5%, and hepatosplenic abscess 1% [34].

The diagnosis of hepatic abscesses in neonates is elusive as the symptoms can be vague and/or non-specific. Therefore, a high index of suspicion is necessary in order to achieve a timely diagnosis and treatment. Common laboratory findings include leukocytosis, thrombocytopenia, elevated acute phase reactants, and altered hepatic enzymes, although jaundice is rare [7]. Radiological findings that should alert the clinician are elevated hemidiaphragm, ipsilateral pleural effusion, and the appearance of enclosed air inside the liver on an X-ray [35]. All neonates with culture-proven fungaemia, as well as those with prolonged sepsis not responding to conventional treatment (especially if umbilical lines remain in situ), should be evaluated for the possible presence of localized lesions such as liver abscess.

Ultrasonography is the preferred imaging modality in neonates for the evaluation of lesions in solid organs since it lacks ionizing radiation, while it is also a pain-free procedure without any need for patient sedation. Moreover, the advent of portable ultrasound machines allows for bedside imaging in the NICU setting. Unfortunately, there are no pathognomonic signs for hepatic abscesses. These lesions usually appear as areas of increased echogenicity, although they can be hypoechoic depending on the chronicity of the infection [15]. Although fungal abscesses have been frequently reported to present as multiple small abscesses scattered in the hepatic parenchyma [36], in most of the neonates included in our study, these lesions presented as solitary lesions. In case of solitary hepatic abscess, the right lobe is more commonly affected due to its larger volume and the increased amount of blood it receives since the flow from the superior mesenteric vein goes preferentially to the right [37,38]. Monitoring with serial ultrasonography is needed in order to evaluate for possible complications, and to assess the response to treatment [7]. Further imaging with CT or MRI can provide more detailed information regarding the location and the extend of the lesion, and can be valuable, especially in case of preoperative planning [12].

Although several medications and interventions have been used for the treatment of neonatal liver fungal abscesses, the optimal therapeutic approach remains debatable. Three classes of antifungal agents are commonly used for the treatment of neonatal fungal infections [39]; polyenes, azoles, and echinocandins. Unfortunately, studies delineating the pharmacokinetics of these medications in the neonatal population are lacking. AMB has been extensively used in the NICU setting due to its favorable safety profile and good tolerance in neonates. Fluconazole, a first-generation triazole, is an alternative agent that is commonly used due to its high efficacy in Candida isolates, its excellent cerebrospinal fluid penetration, and its urinal excretion as an active drug resulting in high concentrations [40]. However, newer antifungal agents such as micafungin and caspofungin have gained ground over recent years, especially in cases where treatment with the traditional agents is precluded due to toxicity or drug resistance [41]. Although *C. albicans* isolates maintain a low incidence of fluconazole resistance (0.5–2%), other species such as *C. parapsilosis* and *C. glabrata* display increasing resistance patterns (2–6% and 11–13%, respectively) or are innately resistant, as is the case with *C. krusei* [42]. Additionally, the emergence of *C. auris*, resistant to azoles (more than 90% of isolates), AMB, and even to echinocandins, poses new challenges given the limited antifungal arsenal at our disposal [43]. Developing antifungal resistance is a growing public health emergency, especially in vulnerable populations, such as neonates. Combined efforts to understand the mechanisms of antifungal resistance and to implement antifungal stewardship programs is of the utmost importance in order to combat this new threat.

Even though conservative treatment with antifungal agents is the first line of treatment of fungal liver abscesses, refractory cases may require invasive interventions such as percutaneous aspirations and/or surgical treatment for the successful resolution of the infection [39]. This approach is feasible only in solitary abscesses and remains challenging in neonates, especially in those born prematurely and with an extremely low birth weight. Among the neonates included in our review, four required abscess drainage, one by open surgical procedure and three by percutaneous placement of a pigtail catheter. Interestingly, in one of these cases, intralesional administration of AMB was additionally performed, resulting in the successful resolution of the infection.

Antifungal prophylaxis in preterm neonates is an area of ongoing controversy, with each institution following a different protocol. Prophylactic fluconazole and oral nystatin have both proven to be successful in the prevention of neonatal IFI. Although there is robust evidence supporting the effective and safe use of fluconazole as antifungal prophylaxis, concerns regarding the potential development of antifungal resistance limits its universal use [44]. A recent nationwide retrospective study in Japan reported that only 43% of medical facilities routinely prescribed antifungal prophylaxis for high-risk neonates [45]. It is noteworthy that none of the neonates included in our review had received antifungal prophylaxis.

Fungal liver abscesses are associated with a high mortality rate in this vulnerable population. Although the data are limited, previous studies have estimated the mortality rate of liver abscess due to *Candida* spp. to be at about 50% [14]. However, the mortality rate in our study was significantly lower (20%), probably reflecting the recent advances in imaging technologies and antifungal agents which result in prompt diagnosis and more effective treatment strategies.

This study has certain limitations. Since a fungal liver abscess is a very rare clinical condition in neonates and the relevant data from the literature are scarce, only case reports and case series were assessed for our systematic review. When searching the literature, no randomized clinical trials nor observational studies were found, and therefore the risk of bias could not be avoided. The results of our study may not be representative of all of the neonatal population due to heterogeneity of clinical presentation, type of treatment, and evaluation of the outcome, and thus they should be interpreted with caution.

## 5. Conclusions

Fungal liver abscess in the neonatal population is a rare clinical entity, with only 19 reported cases in the literature. Due to the non-specific clinical and laboratory findings, diagnosis is challenging as it requires a high index of suspicion, especially in neonates with predisposing risk factors such as prematurity, umbilical vein catheter placement, prolonged administration of broad-spectrum antibiotics, and total parenteral nutrition. Although these infections are severe and are associated with a high mortality rate, recent advances in imaging have facilitated a prompt diagnosis, resulting in improved eradication rates. A multidisciplinary approach with the collaboration of neonatology, interventional radiology, and surgery experts is necessary to provide the best available care for this vulnerable population.

## Figures and Tables

**Table 1:** Characteristics of individual studies.

Study	Patients	GA (Weeks)	BW (g)	Gender	Diagnosis Day of Life	Fungal Species	Isolated from	Location	Other Location	Underlying Condition	Clinical Manifestations	Diagnosis	Concomitant Bacteria	Radiological Findings	Type of Treatment	Treatment Duration (Days)	Antifungal Therapy	Outcome
Noyola 2001, [11]	1	26	979	NR	NR	*Candida albicans*	Blood, CSF	NR	NO	Prematurity, central line catheter	NR	Abdominal U/S	NO	NR	Antifungal therapy	30	d-AMB (1 mg/kg, for 30 days)	Survived
	2	25	990	NR	NR	*Candida albicans*	Blood	NR	NO	Prematurity, central line catheter	NR	Abdominal U/S	NO	NR	Antifungal therapy	5.5	d-AMB (1 mg/kg, for 6 days)	Death attributed to Candida infection.
Tan 2005, [4]	1	24	960	Male	19	*Candida* spp.	Blood, CSF	Right lobe	Cerebral abscesses	Prematurity, central line catheter	Sepsis, hypotension	Hepatobiliary system U/S	*Acinetobacter baumannii*	Multiple liver microabscesses	Antifungal and ampicillin/sulbactam therapy	59	AMB and flucytosine (28 days)	Death
	2	34	1900	Female	33	*Candida glabrata*	Blood, liver abscess culture	Right lobe	NO	Prematurity, surgery for tracheoesophageal fistula and duodenal atresia	Symptomatic adhesions	Operative finding	*MRSA*	NR	Open drainage of abscess and antifungal therapy	42	d-AMB (42 days)	Survived
	3	24	660	Male	11	*Candida albicans*	Blood, pleural fluid culture	Right lobe	Cerebral abscesses, right empyema thoracis	Prematurity, central line catheter, sepsis, NEC	Sepsis	Hepatobiliary system U/S	*Klebsiella* spp.	Tiny echogenic foci (microabscesses)	Ampicillin/gentamicin and amikacin/cefotaxime.	11	No antifungal therapy*(blood culture positive for Candida after the patient expired)	Death
Cascio, 2014, [12]	1	33	1950	Female	11	*Candida albicans*	Blood, tip of UVC catheter	Fifth and seventh hepatic segments	NO	Prematurity, central line catheter, sepsis	Sepsis	Abdominal U/S	NO	Two rounded focal areas with a hypoechoic content and peripheral hyperechoic rim	Antifungal therapy	32	L-AMB (3 mg/kg/day, for 32 days)	Survived
	2	33^+4^	2250	Male	10	*Candida albicans*	Blood, tip of UVC catheter	Third and fourth hepatic segments	NO	Prematurity, central line catheter, sepsis	Sepsis, vomiting	Abdominal U/S and CT	NO	A pseudonodular area with a mature echogenic wall and heterogeneous semi-solid contents with areas of central necrosis	Antifungal therapy	40	L-AMB (3 mg/kg/day, for 40 days)	Survived
	3	34	2380	Male	28	*Candida albicans*	Blood, tip of UVC catheter	Third and fourth hepatic segments	NO	Prematurity, central line catheter, sepsis, broadspectrum antibiotics	Sepsis	Abdominal U/S	NO	Rounded focal hyperechoic area (diameter, 30 mm)	Antifungal therapy	14	Fluconazole (6 mg/kg/day, for 4 days), then L-AMB (3 mg/kg/day, for 30 days)	Survived
Abdeljelil, 2012, [13]	4 (CASES)	NR	NR		18.5 ± 6.9 days (range 6 to 42 days)	*Candida* spp.	Blood, liver abscess culture	NR	NR	Prematurity, central line catheter, sepsis	NR	NR	NR	NR	Antifungal therapy	NR	NR	NR
Picone, 2013, [14]	1	31	1690	Male	5	*Candida albicans*	Blood	Right lobe	Hyperechogenic endoluminal mass in the gallbladder	Prematurity, central line catheter, sepsis	Sepsis	Abdominal U/S	NO	A multiloculated, 3-cm diameter liver abscess	Antifungal therapy	38	Micafungin (3 mg/kg/day, for 38 days)	Survived
Sharma, 2015, [15]	1	30	1400	Male	25	*Candida albicans*	Blood, liver abscess culture	Segment VI of the liver	NO	Prematurity, central line catheter, malpositioned UVC inside theliver	Sepsis	Abdominal U/S	NO	Hyperechoic, thick-walled, 60|25|40 mm collection	Antifungal therapy and abscess drained using a pigtail drainage catheter	4 weeks	d-AMB (4 weeks)	Survived
Auriti 2018, [16]	1	28	1000	NR	8	*Candida albicans*	Blood, peritoneal fluid culture, liver abscess culture	Right lobe		Prematurity, central line catheter, TPN	Sepsis, hypotension	Abdominal U/S and CT	NR	Multilobulated intrahepatic abscess	Antifungal therapy and abscess drained using pigtail catheter and intralesional AMB	28	Micafungin IV and AMB IV and intralesional (total 28 days)	Survived
Filippi 2009, [17]	1	25^+5^	820	Female	13	*Candida albicans*	Blood	NR	NR	Prematurity, UVC with tip in the liver used for TPN, ileal atresia	Sepsis	Abdominal U/S	NR	Multiple liver abscesses (4)	Antifungal therapy	77	Fluconazole (6 mg/kg/48 h, for 77 days), L-AMB (3 mg/kg/day, for 59 days), Caspofungin (1 mg/kg/day for 46 days)	Survived
	2	35^+2^	2250	Male	85	*Candida albicans*	Blood, bronchoalveolar lavage	NR	NR	Prematurity, prolonged intubation/MV, wide ASD & VSD-PA banding, chromosomal anomaly	NR	Abdominal U/S	NR	Multiple (miliary) liver abscesses	Antifungal therapy	53	Fluconazole (6 mg/kg/day, for 33 days) then Caspofungin (5 mg/kg/day, for 20 days)	Survived
Cantey 2020, [18]	1	Term	4960	Female	4	*Malassezia* spp.	Calcofluor whitestain of liver abscess fluid, negative cultures of blood and liver abscess	Left lobe	NR	Gestational diabetes mellitus, malpositioned UVC used to infuse lipids	Sepsis, fever, liver mass	Abdominal U/S and CT	NR	Multicystic liber mass/abscess	Antifungal therapy and abscess drained using pigtail catheter	28	d-AMB (1 mg/kg/day, for 28 days)	Survived
Doerr 1994, [19]	1	26	895	Male	30	*Candida parapsilosis*	Subphrenic abscess fluid culture	Right lobe	Subphrenic abscess (right)	Prematurity, NEC with perforation	Abdominal distention	Abdominal U/S and CT	*Xanthomonas maltophilia*	Mass with variable consistency and fluid/air levels	Antifungal and antimicrobial therapy	NR	AMB (30 mg/kg)	Survived

Abbreviations: amphotericin, AMB; gestational age, GA; birth weight, BW; cerebrospinal fluid, CSF; computed tomography, CT; methicillin-resistant Staphylococcus aureus, MRSA; not reported, NR; total parenteral nutrition, TPN; umbilical venous catheter, UVC; ultrasonography, U/S.

**Table 2:** Demographics of the study population.

Variables	Neonates (n = 19)
Gestational Age (weeks)	29.0 (24.0- term)
Patient age (days)	22.5 (7.0–59.0)
Gender (male)	8/12 (66.6)
Predisposing factors Prematurity Total parenteral nutrition administration Umbilical venous catheter placement Mechanical ventilation Broad-spectrum antibiotic administration	14/15 (93.0) 12/13 (92.3) 13/13 (100.0) 7/8 (87.5) 11/12 (91.7)

Data are presented as medians and ranges, or as absolute frequencies (percentages) when appropriate.

**Table 3:** Microbiology of fungal species and co-cultured bacterial pathogens.

Genus	Pathogen	Patients (n = 19)
*Candida* spp.	*C. albicans*	11 (57.9)
	*C. parapsilosis*	1 (5.2)
	*C. glabrata*	1 (5.2)
	*Unidentified Candida* spp.	5 (19.0)
*Malassezia* spp.	*Unidentified Malassezia* spp.	1 (5.2)
Co-cultured bacterial pathogens	*Staphylococcus* spp.	1 (5.2)
	*Acinetobacter baumanii*	1 (5.2)
	*Klebsiella* spp.	1 (5.2)
	*Xanthomonas maltophilia*	1 (5.2)

Data are presented as absolute frequencies (percentages).

**Table 4:** Diagnosis of fungal infections.

Diagnostic Modality	Neonates (n = 19)
Positive histological findings	1 (5.2)
Positive culture Blood specimen Abscess fluid specimen Cerebrospinal fluid specimen	17/19 (89.5) 8/19 (42.1) 2/19 (10.5)
Ultrasonography Solitary lesion Multiple lesions	8/12 (66.7) 4/12 (33.3)

Data are presented as absolute frequencies (percentages).

**Table 5:** Treatment strategies and survival rate.

Parameters	Patients (n = 19)
* Antifungal therapy AMB monotherapy AMB and flucytosine Fluconazole followed by AMB Fluconazole followed by caspofungin Micafungin Micafungin and AMB Fluconazole, AMB and caspofungin	8/15 (53.0) 1/15 (6.6) 1/15 (6.6) 1/15 (6.6) 1/15 (6.6) 1/15 (6.6) 1/15 (6.6)
Invasive intervention Percutaneous pigtail catheter Open surgical drainage	4 (21.0) 3 (15.7) 1 (5.2)
** Survived	12 (80.0)

Data are presented as absolute frequencies (percentages) when appropriate. * Median duration of treatment (days) was 34.2 (range: 28.0–42). ** Data on the outcome were not available in 4 cases.

## Data Availability

Data are contained within the article.

## Supplementary Materials

The following supporting information can be downloaded at: https://www.mdpi.com/article/10.3390/life13010167/s1. Reference [46] has cited in Supplementary Materials.
